# New Insights on the Formation of the Mitral Valve Chordae Tendineae in Fetal Life

**DOI:** 10.3390/jcdd11110367

**Published:** 2024-11-15

**Authors:** Meghan Martin, Kate Gillett, Parker Whittick, Sarah Melissa Wells

**Affiliations:** 1 School of Biomedical Engineering, Dalhousie University, Halifax, NS B3H 4R2, Canada; meghanmartin@dal.ca; 2 Department of Biochemistry and Molecular Biology, Faculty of Medicine, Dalhousie University, Halifax, NS B3H 4R2, Canada; kt995186@dal.ca; 3 Medical Sciences Program, Faculties of Science and Medicine, Dalhousie University, Halifax, NS B3H 4R2, Canada; pr703389@dal.ca

**Keywords:** heart valves, extracellular matrix, chordae tendineae formation, valvular endothelial cells, matrix metalloproteinases, valvulogenesis, elastin, collagen, cardiac development

## Abstract

There is an increasing understanding that some mitral valve pathologies have developmental origins. The time course of valvulogenesis varies by animal model; in cattle, the branched chordae tendineae architecture becomes fully developed at full term. The mechanism by which chordae tendineae bifurcate during fetal development remains unknown. The current study presents a detailed description of bovine chordae tendineae formation and bifurcation during fetal development. Analysis of Movat Pentachrome-stained histological sections of the developing mitral valve apparatus was accompanied by micro-CT imaging. TEM imaging of chordae branches and common trunks allowed the measurement of collagen fibril diameter distributions. We observed a proteoglycan-rich “transition zone” at the junction between the fetal mitral valve anterior leaflet and chordae tendineae with “perforations” lined by MMP1/2 and Ki-67 expressing endothelial cells. This region also contained clusters of proliferating endothelial cells within the bulk of the tissue. We hypothesize this zone marks a region where chordae tendineae bifurcate during fetal development. In particular, perforations created by localized MMP activity serve as a site for the initiation of a “split” of a single chordae attachment into two. This is supported by TEM results that suggest a similar population of collagen fibrils runs from the branches into a common trunk. A clear understanding of normal mitral valvulogenesis and its signaling mechanisms will be crucial in developing therapeutics and/or tissue-engineered valve replacements.

## 1. Introduction

**Figure 1:**
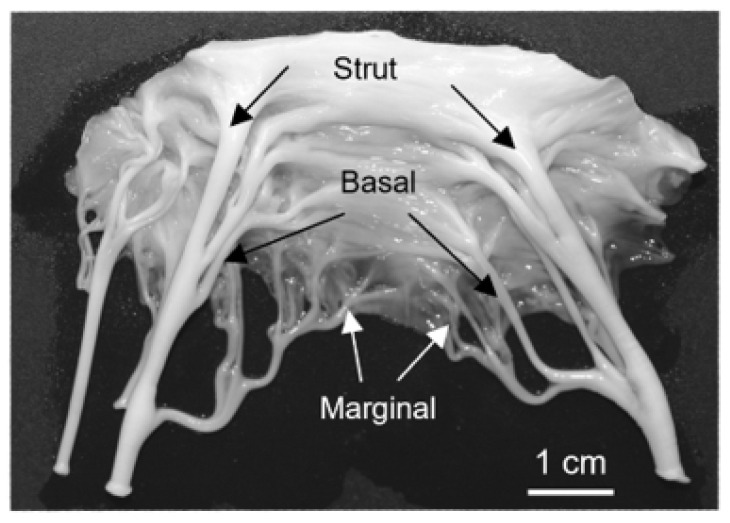
Representative image of an excised adult bovine anterior mitral valve. Arrows denote (in order from top to bottom) the strut, basal, and marginal chordae.

The proper function of the mitral valve is essential for maintaining unidirectional blood flow through the heart. The valve consists of four main components: the annulus, anterior and posterior leaflets, papillary muscles, and chordae tendineae [1]. The chordae tendineae connect the leaflets to the papillary muscles [2,3] and are classified based on their size and where they attach to the leaflet [4,5]. The thinnest marginal chordae attach to the free edge of the leaflet, while the intermediately sized basal chordae attach directly to the ventricular surface of the leaflet [5]. The thickest strut chordae attach near the midline of the leaflet around the 4 o’clock and 8 o’clock positions [4,5]. Chordae tendineae exhibit a branching pattern, with a single chord from the papillary muscle bifurcating into thinner strands and creating a fan-like network of attachments into the leaflet (Figure 1). The chordae tendineae prevent the prolapse of the leaflet into the atrium by transferring tension from the leaflet to the papillary muscles [2,3], with most of this tension borne by the strut chordae [6].

Chordae tendineae are tendinous tissues comprised of collagen, elastin, proteoglycans, and valvular interstitial cells. The exterior of the chordae tendineae is lined by a layer of endothelial cells underneath, which is a layer of elastin interwoven with a small amount of loosely bound fibrous collagen [7]. The inner core is composed of tightly bundled fibrous collagen, which makes up 60% of the chordae dry weight and contributes to the impressive tensile strength of these structures [8,9].

Proper functioning of the anterior mitral valve leaflet is essential for maintaining the unidirectional blood flow from the left atrium to the left ventricle, and any damage to the chordae tendineae can disrupt this process. Diseases or dysfunction of the chordae tendineae, such as rupture, elongation, or fibrosis, can result in mitral valve prolapse or regurgitation [10,11,12]. Current treatment for mitral valve diseases includes surgical repair or valve replacement. In repair, chordae can either be resected, shortened, or replaced with neochordae [13]. While increasing evidence shows that chordal replacement may be more advantageous, the long-term mortality [13] and rates of reoperation [14] remain similar between resection and replacement. Currently, there are no tissue-engineered chordal replacements on the market. The main issue in creating tissue-engineered replacements is our lack of understanding of chordae developmental mechanisms. Therefore, identifying these mechanisms will contribute to the development of tissue-engineered chordae tendineae.

Important as they are for mitral valve function, there remains no comprehensive explanation of how chordae tendineae form and bifurcate during fetal development. What has confounded chordal development work is that this process appears to be species-dependent. In rats and mice, anterior leaflet chordae tendineae do not bifurcate until after birth [15,16,17,18,19]. Specifically in rats, branched chordae do not appear until approximately seven days after birth [15]. Mice possess one to two unbranched chordae tendineae by late gestation [16,19] and up to 4 chordal tendons at birth. These tendons undergo several generations of branching postnatally, forming an average of fifteen attachments into the leaflet in adulthood [17]. The lack of branched chordae during rodent gestation may be due to the small valve orifice and low ventricular pressures [15]—such that the primordial leaflet alone can provide adequate valve closure. As the valve orifice expands and ventricular pressure increases after birth, the chordae tendineae bifurcate into multiple attachment sites, providing extra support to the leaflet and preventing prolapse [15,16].

By contrast, in large mammals, such as pigs [20], humans [21,22], and cattle [23], chordae tendineae form and bifurcate *during gestation*. In humans, branched chordae tendineae are seen as early as ~89 days into gestation, before the anterior leaflet has fully delaminated from the underlying myocardium [21,22]. In our previous studies on cattle, the number of chordae attachments to the growing anterior leaflet increases throughout gestation. The number of chordae tendineae attachments is then fixed at birth despite the continued growth of the leaflet post-partum [23]. These observations suggest that there may be a programmed geometry and/or number of chordae tendineae attachments that are optimal for adult mitral valve function and that this geometry is reached by birth. Thus, it is important to study chordae formation during gestation in order to discover the underlying developmental mechanisms.

Some clues regarding the signaling mechanisms underlying chordae formation have come from genetic knockout studies in mice. The matricellular protein periostin appears necessary, as periostin knockout mice do not develop branched chordae tendineae after birth. Tricuspid leaflets in periostin null adult mice exhibit the same number of non-branched chordae as wildtype neonates (*n* = 4) [17]. There appears to be a mechanical signaling component to chordae formation as filamin-A, a cytoskeletal protein, is also required. Mice lacking filamin-A possess two large chordae with no branching, similar to the phenotype of the mice lacking periostin [24].

Yet, the question remains: how do the chordae form and bifurcate? Some of the first theories of chordae tendineae formation were drawn from serial sections of fetal human atrioventricular valves. In these models, it was hypothesized that the chordae are derived from myocardial tissue [25,26]. However, lineage tracing studies demonstrated that the chordae and leaflets are each derived from distinct populations of endocardial cells [18]. Later studies in humans [21], rats [15], and chicks [27] confirmed that chordae are derived from the endocardial tissues. The endocardial cushions are remodeled from a gelatinous cushion to a thinner sheet attached to the myocardium before delamination. Narrow slits or “gaps” in this tissue preceded the appearance of branched chordae tendineae [21,27]. It was initially proposed that gap formation was caused by apoptosis of leaflet cells, similar to the process underlying its delamination from the myocardium [18]. This theory was later discredited as it was demonstrated that minimal apoptosis was occurring in these areas [28,29,30].

The goal of the present study was to evaluate the possible mechanisms of chordae formation in a large mammalian model (cattle) where chordae form during gestation. Cattle are an ideal model in that they share with humans a similar four-chambered heart [31], mitral valve anatomy [23], transvalvular pressures, and a 9 month gestation period. Here, we report the first comprehensive study of chordae tendineae formation in a large animal model. We observed a “transition zone” at the junction between the fetal mitral valve leaflet and chordae tendineae, featuring a proteoglycan-rich area with holes or “perforations” lined by MMP-1, MMP-2, and Ki-67 expressing endothelial cells. By late gestation, there is a differential remodeling of elastin and collagen, with elastic fibers accumulating circumferentially around the perforations and collagen fibers aligning parallel to the chordae tendineae. We hypothesize that the transition zone region marks the origin of new chordae branching, where perforations form, direct ECM organization, and initiate the splitting of single chordae attachments into two.

## 2. Materials and Methods

### 2.1. Tissue Harvest and Anatomical Dimensions

Protocols for the harvesting of bovine tissues (*Bos taurus*) were approved by the University Committee on Laboratory Animals at Dalhousie University. Fetal bovine hearts from both males and females were obtained from a local abattoir immediately following slaughter [23]. To determine gestational age, the fetal crown to rump length was measured using the following equation [32]:

Gestational Day = 8.4 + 0.087(CRL) + 5.46(CRL)^1/2^

Fetuses ranged in age from 68 days (9 cm fetal length) to 270 days (100 cm fetal length). The anterior leaflet and associated chordae tendineae were excised before undergoing their respective fixation process for each method. Consistent with previous studies [23], this work focuses on the anterior leaflet as it experiences higher stresses in vivo and as the larger leaflet has more tissue available for experimentation.

### 2.2. Histological Preparation

One-half of the mitral valve anterior leaflet with associated chordae tendineae was cut from fetal hearts in the second and third trimesters (91 to 270 days). However, for first trimester samples (0 to 90 days), the whole anterior leaflet and chordae were used due to their small size. Samples were fixed (10% buffered neutral formalin) for 7 days, embedded in paraffin, and sectioned into 4 µm serial transverse sections. For immunohistochemistry, negative controls were run with each batch of staining.

### 2.3. Movat Pentachrome Staining

Sections were stained with the Russell–Movat Pentachrome Stain Kit (Statlab, McKinney, TX, USA) to determine the localization of ECM proteins using the manufacturer’s instructions. This kit stains collagen yellow-orange, mature elastin dark purple, muscle tissue and blood cells red, glycosaminoglycans in blue-green, and cell nuclei in dark red-purple. Movat stained slides were imaged using a Pannoramic MIDI II and viewed with the Caseviewer software (Version 2.4, 3DHistech, Budapest, Hungary).

### 2.4. Picrosirius Red Staining

To examine collagen alignment in the transition zone, sections were stained with picrosirius red and hematoxylin. Images of picrosirius-red–stained sections were taken using a Nikon Eclipse E600 light microscope (Nikon Instruments Inc., Melville, NY, USA) equipped with a polarizer and an AmScope 10MU1400 digital camera (AmScope, Irvine, CA, USA) to better visualize collagen.

### 2.5. Immunohistochemistry

Antibody staining followed similar steps, with some variations for each antibody. Slides were subjected to heat induced epitope retrieval with Tris-EDTA (pH = 9) at 95 °C (time varied between antibodies, Table 1) before a peroxide block for 5 min. Immunohistochemistry for each antibody was conducted on a Bond RX Automated IHC Stainer (Leica, Concord, ON, Canada), using the primary antibody (Table 1) and Bond Polymer Refine Detection (Leica, cat no DS9800). Staining was carried out according to the manufacturer’s protocol with some modifications (Table 1). For CD31 and MMP-2, a tissue blocking step was added prior to the incubation in the antibody solution by applying PowerVision IHC/ISH Super Blocking (Leica, cat no PV6122) to the tissues for 30 min. Primary antibody incubation times, polymer solution incubation, and DAB incubation varied between antibodies (Table 1). The solution for primary antibody incubation contained both the primary antibody (dilutions in Table 1) and Bond Primary Antibody Diluent (Leica cat no AR935). Slides were then incubated in polymer solution (polymeric HRP-linker anti-rabbit IgG antibody conjugates) and washed two times with Bond Wash Solution and a wash with purified water. This was followed by incubation with DAB, three washes with purified water, and Haematoxylin counterstaining for 5 min. Slides were then dehydrated in a series of ethanol and coverslipped in a Spectra Workstation (Leica, Concord, ON, Canada). Slides were scanned using an Aperio XT Brightfield Whole Slide Scanner (Leica, Concord, ON, Canada) at 400 times magnification.

### 2.6. Cell Density Measurement

The presence of Ki-67 positive nuclei was assessed in the anterior leaflet, the chordae tendineae, and the transition zone. Photo analysis grids with an area of 0.64 mm^2^ were overlaid onto the image of the whole valve (taken at 1.5X). Three random grids were chosen per area for analysis, for a total of nine grids per valve. Grid areas were enhanced to a magnification of 13.6X for further analysis in ImageJ (National Institute of Health, Bethesda, MD, USA). The Cell Count plugin feature in ImageJ was used to manually count and classify cell nuclei as either Ki-67 positive (which appears brown with DAB staining) or negative (which appears purple/blue with hematoxylin counterstain). First, all cell nuclei in the image (positive and negatively stained cells) were selected and recorded. Next, only the count Ki-67 positive cell nuclei in the image were recorded. Both total cell number and Ki-67 positive cell numbers were normalized to the area of the grid to get total cell density and Ki-67 positive cell density, respectively.

### 2.7. Microcomputed Tomography Preparation, Imaging, and Volume Rendering

Fetal valves for microcomputed tomography (micro-CT) imaging ranged in age from 125 to 236 days gestation. The whole anterior leaflet, associated chordae tendineae, and papillary muscle attachments were dissected and fixed with 4% paraformaldehyde overnight. Tissues were then placed into a solution of 10% Lugol’s iodine for 7 days in the dark. Following this, tissues were rinsed with distilled water and placed into a phosphate buffered saline solution at 4 °C in the dark until imaging.

Micro-CT images were taken using the Bruker SkyScan 1276 Micro-CT (Bruker, Billerica, MA, USA). To accommodate the small tissues, the carrier was wrapped in parafilm. The anterior mitral apparatus was left in PBS until being placed onto the parafilm covered carrier ventricular side facing up. To prevent desiccation during scanning, PBS was gently dripped onto the samples. The samples were then scanned for 30 min with parameters optimized for ex vivo imaging (58 KV, 200 µA, and voxel size of approximately 9 µm). Three-dimensional reconstruction and volume rendering were performed using Dragonfly 4.1 (Object Research Systems (ORS), Montreal, QC, Canada). Three-dimensional renderings were sliced through manually using the clipping tool, and single 2D images were exported in Tag Image File Format (TIFF).

### 2.8. Perforation Measurement

**Figure 2:**
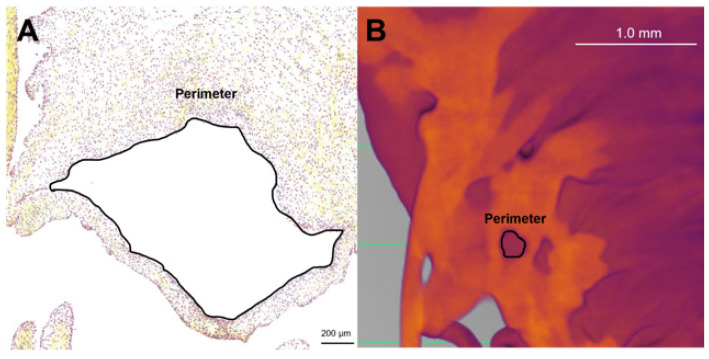
Methods for measuring perforation dimensions in the two modalities. The perimeter was measured as an approximation of perforation size. Representative image of these measurements in a Movat Pentachrome stained section from 219 gestational days (**A**). Representative image of these measurements in 2D still image from micro-CT from 163 gestational days (**B**). Note the scale bar varies between images.

To determine if the perforations visualized within the transition zone in both histology and micro-CT were similar, the size of the perforations was compared. Images taken from both modalities were uploaded to ImageJ (Version 1.54k, ImageJ, National Institutes of Health, Bethesda, MD, USA), and the free hand tool was used to trace the perimeter (Figure 2). All visible perforations in an image were measured. For histology images, valves from a total of 13 animals ranging from 111 gestational days to 270 gestational days were measured. For micro-CT images, valves from a total of 5 animals ranging from 148 gestational days to 236 gestational days were measured.

### 2.9. Transmission Electron Microscopy

**Figure 3:**
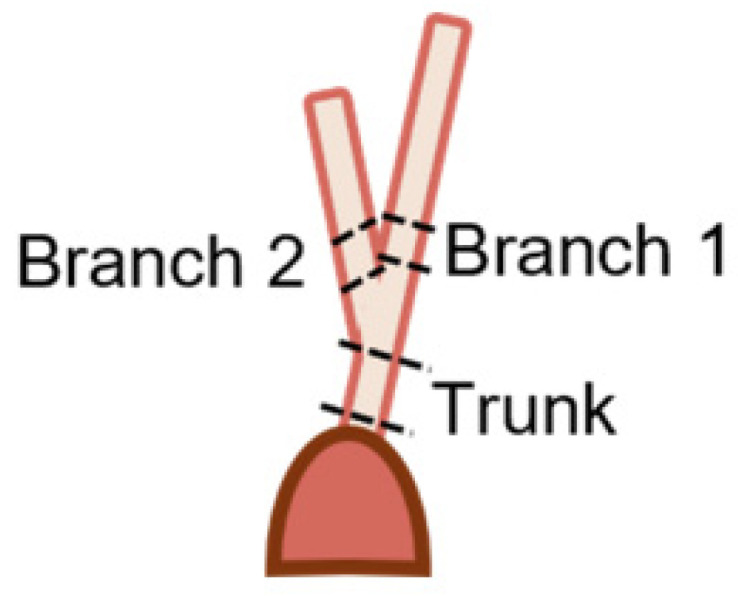
Diagram depicting the location of samples for transmission electron microscopy. Trunk sections were taken from above the papillary muscle connection to just below the first bifurcation. Branch sections were taken from above the bifurcation to below their next bifurcation.

Fetal samples collected for transmission electron microscopy (TEM) imaging of collagen fibril diameters ranged from 68 to 270 days (full term). To ensure consistent anatomical location and mechanical loading conditions, we harvested the strut chordae and its two daughter branches from the papillary muscle attachment to the first bifurcation (Figure 3). Sample lengths varied depending on gestational age. Samples were rinsed in PBS three times before being fixed with 2.5% glutaraldehyde solution (Electron Microscopy Sciences, Hatfield, PA, USA), diluted with 15 mL of 2.0 M sodium cacodylate buffer (Electron Microscopy Sciences, Hatfield, PA, USA), and 15 mL of deionized water overnight.

The following day, samples were taken to the Electron Microscopy Core Facility at Dalhousie University, where the remainder of the preparation took place. After the initial fixation, samples were rinsed four times in 0.1 M sodium cacodylate buffer for 10 min each. Next, the samples were fixed again with 1% osmium tetroxide (Alfa Chemistry, Holbrook, NY, USA) for two hours and then rinsed in distilled water. The samples were then placed in 0.25% uranyl acetate (Fisher Scientific Company, Ottawa, ON, Canada) at 4 °C and left overnight. Following this, samples were dehydrated in a graduated series of acetone at 50%, 70%, 95%, and 100%. Samples were then infiltrated with Epon Araldite Resin (Electron Microscopy Sciences, Hatfield, PA, USA), first with a 3:1 ratio of acetone (Fisher Scientific Company, Ottawa, ON, Canada) to resin for 3 h, then a 1:3 ratio of acetone to resin overnight, and finally in 100% resin two times for 3 h each. Samples were then embedded in the resin and heated in an oven at 60 °C overnight. Sections of approximately 100 nm thick were cut using a Reichert–Jung Ultracut E Ultramicrotome (Depew, NY, USA) with a diamond knife and placed on TEM copper grids (300 mesh). The grids were stained with 2% aqueous uranyl acetate, rinsed twice with distilled water, and then stained with lead citrate (TAAB Laboratories, Berks, UK). They were then rinsed one final time with distilled water and allowed to air dry.

### 2.10. Collagen Fibril Diameter Measurement

Samples were viewed using a JEOL JEM 1230 Transmission Electron Microscope (JEOL, Tokyo, Japan) at 80 kV equipped with a Hamamatsu ORCA-HR digital camera (Hamamatsu Photonics, Shizuoka, Japan) and images were captured at 50,000 times magnification. For each section (trunk, branch 1, and branch 2), three of the TEM grids were assessed, and three images were taken per TEM grid for a total of nine images per section. Diameter measurements were made in ImageJ (ImageJ, National Institutes of Health, Bethesda, MD, USA), where an image analysis grid with an area of 7.04 × 10^4^ nm^2^ was overlaid on the images. Then, only fibrils lying along the gridlines, possessing a circular shape and discernable outer boundaries, were measured. Using the oval tool, the largest possible circle that did not extend outside the fibril’s visible perimeter was fit to each fibril, and the area of the circle was recorded. Fibril diameter was then calculated using the equation:

$$Diameter=\left(\sqrt{\frac{Area}{\pi }}\right)*2$$

**Figure 4:**
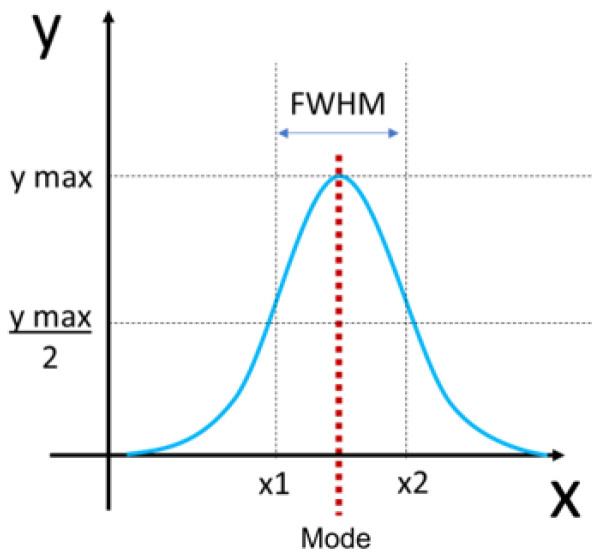
Determination of full width at half maximum (FWHM) and mode calculation from collagen fibril diameter distributions. The FWHM was measured by first identifying the maximum value of the distribution (y max) and then dividing that by 2 (y max/2). To find x1 and x2, the x value that corresponded with the maximum y value was defined. This was then used as a threshold to find the minimum (x1) and maximum value (x2) of x that corresponded with y max/2. FWHM was calculated by subtracting x2 minus x1 (blue double-sided arrow). The mode was defined as the most common value in the distribution (red dotted line).

For each chordae section (trunk, branches), the fibril diameter histogram was produced, and the mode and full width at half maximum (FWHM) were determined (Figure 4). Histograms between the two branches were merged as they were not significantly different.

### 2.11. Fibril Density

Fibril density was also measured in ImageJ (ImageJ, National Institutes of Health, Bethesda, MD, USA) from the same images used to measure diameter. The multipoint tool was used to count the number of fibrils in each image and total fibril number was normalized to total image area (7.97 × 10^6^ nm^2^) to give density.

### 2.12. Statistical Analysis

To compare the perforation size (as perimeter) between light microscopy and micro-CT modalities, a Mann–Whitney U test was conducted (as the distribution for each modality was nonnormal). A significant difference was considered when *p* < 0.05.

From the distributions of collagen fibril diameters, modes were calculated in R (2023.06.0 Build 421) [33] using the modes function from the multimode package [34]. To determine whether the distributions were unimodal or bimodal, Hartigan’s Diptest from the diptest package (Version 0.76.0) [35] was used. Since there was no significant difference between the diameters of the two branch chordae, they were binned together for analysis. The FHWM was calculated in R using a custom-built function.

To assess the changes in each parameter during fetal development, data were plotted as a function of gestational age in days and fitted with a linear least squares regression. In the case of perforation perimeter, a power relationship provided the best fit, and the *p* value was assessed from a linear regression log transformation. The regression was considered significant when *p* < 0.05. To compare the rate of change within any single parameter during fetal development between trunk and branch chordae, the regression slopes were compared using an analysis of covariance (ANCOVA). Differences between the slopes were considered significant when *p* < 0.05. All statistical analyses were conducted using R, specifically the car (Version 3.3.1) [36], mvnormtest (Version 0.1.9) [37], DescTools (Version 0.99.48) [38], devtools (Version 2.4.4) [39], and ggplot2 (Version 3.3.6) [40] packages.

## 3. Results

### 3.1. The Fetal Mitral Valve Anterior Leaflet Contains a Unique, Perforated ‘Transition Zone’

**Figure 5:**
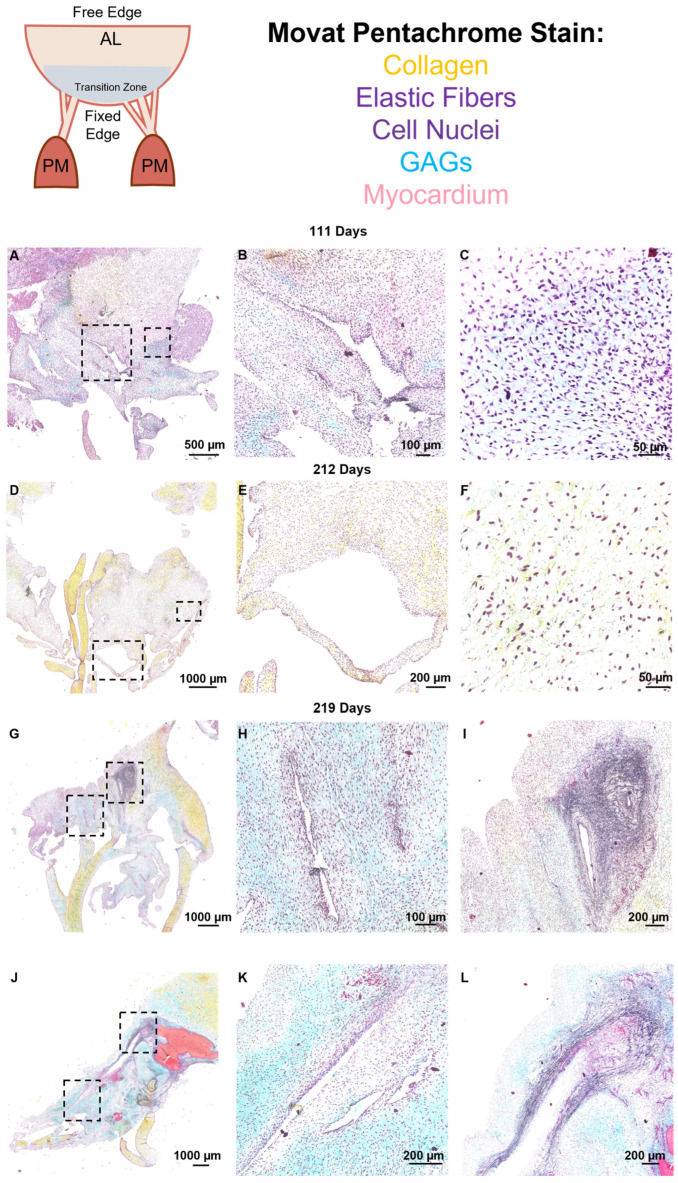
Representative images of the “transition zone” found between the collagenous portions of the mitral anterior leaflet and chordae tendineae throughout gestation. (**A**,**D**,**G**,**J**) Overview of the “transition zone” in the anterior leaflet. Dashed boxes show where subsequent magnified panel images were taken from. (**B**,**E**,**H**,**K**) Magnified images of the cell-lined perforations present in this area. (**C**) Magnified image of the “transition zone” demonstrating a lack of elastic fiber presence at earlier time points. (**F**,**I**,**L**) Magnified images showing the pattern of elastic fiber organization in this transition zone at later developmental stages. (**A**–**C**) valve in the early second trimester (111 days into gestation). (**D**–**F**) third trimester (212 days into gestation). (**G**–**I**) third trimester (219 days into gestation). (**J**–**L**) full term (270 days). The long axes of perforations were often in the direction of the chordae tendineae. (Figure 5G,J and Figure 6B,C). Collagen is stained yellow-orange, elastic fibers are dark purple, muscle tissue, and blood cells are red, glycosaminoglycans are blue-green, and cell nuclei are dark red-purple. The scale bar varies per image.

**Figure 6:**
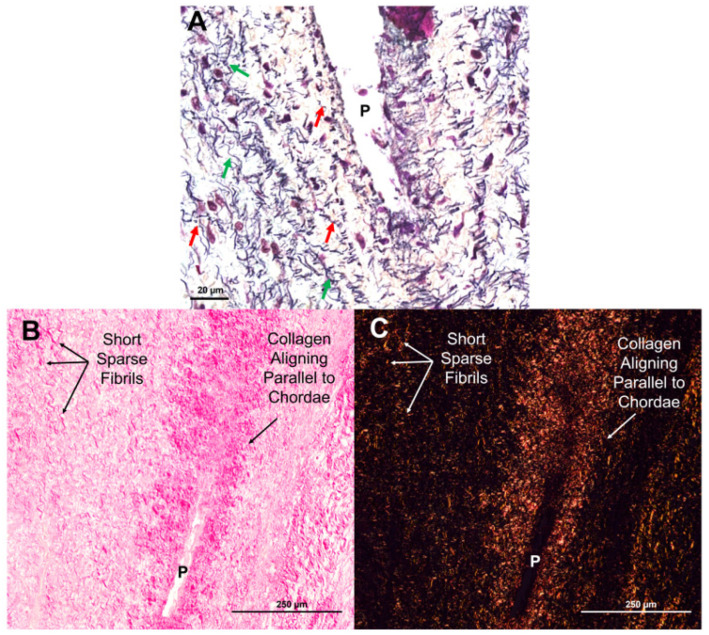
Elastin and collagen fibers first appear as short, sparse fragments before beginning to align in parallel with the chordae. (**A**) Movat pentachrome stained section of a perforation (P) with short elastic fragments (red arrows) and longer fragments (green arrows) beginning to align around it. Image taken at 14X magnification using the Pannoramic MIDI II. (**B**,**C**) Picrosirius red and hematoxylin-stained sections of a perforation (P) with shorter sparse collagen fibrils and collagen beginning to align parallel to the chordae under brightfield (**B**) and polarized light (**C**). Images were taken on a Nikon Eclipse E600 light microscope (Nikon Instruments Inc., Melville, NY, USA) equipped with a polarizer and an AmScope 10MU1400 digital camera (AmScope, Irvine, CA, USA). All photos were taken from sections from the same third trimester valve (219 days into gestation). The scale bar varies per image.

Movat pentachrome staining revealed the ECM organization over the entire fetal anterior mitral valve leaflet and chordae tendineae (Figure 5). Interestingly, separating the circumferentially aligned collagen of the leaflet and the collagenous connections of the chordae tendineae was a large “transition zone” area comprised of predominantly glycosaminoglycans, cells, and cell-lined perforations (Figure 5A,D,G,J). The perforations were distinct from artifacts of sectioning (which contained folds or tears in the tissues with no cells present within the inner lining), having an almost continuous layer of cells. The long axes of perforations were often in the radial direction of the leaflet, i.e., in the direction of the chordae tendineae (Figure 5G,J and Figure 6B,C).

### 3.2. Collagen and Elastic Fibers Coalesce and Rotate Around Perforations

By the early third trimester, sparse elastic fibers were evident in the transition zone (212 days, Figure 5C,F). Short elastic fibers begin to accumulate around perforations, usually aligned in the circumferential direction of the leaflet, perpendicular to the direction of the chordae tendineae (Figure 6A, red arrows). Mature elastic fibers increased in density until full term, mainly in circumferential alignment around perforations (Figure 5I,L). Higher magnification images show the striking contrast between short, circumferentially-aligned elastic fiber fragments (Figure 6A, red arrows) and the longer, radially aligned fragments (Figure 6A, green arrows). A similar pattern was observed with the deposition of collagen in the transition zone: short fragments appearing sparsely early in gestation with increased density and alignment around perforations by late gestation (Figure 5B,E,H,K and Figure 6B,C).

### 3.3. Endothelial Cells Line the Transition Zone Perforations

**Figure 7:**
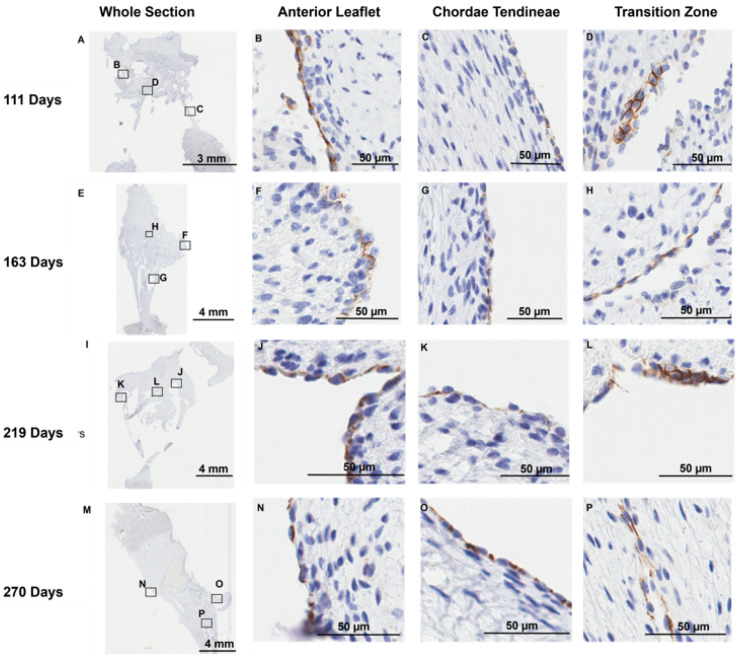
Representative images of CD-31 (endothelial cell marker) expression throughout the mitral valve anterior apparatus. (**A**,**E**,**I**,**M**) Overview of the CD-31-stained sections. Boxes show where subsequent magnified panel images were taken from. (**B**,**F**,**J**,**N**) Magnified images of the outer edge of the anterior leaflet showing CD-31 staining in the cells lining the leaflet. (**C**,**G**,**K**,**O**) Magnified images of the outer edge of the chordae tendineae showing CD-31 staining in the cells lining them. (**D**,**H**,**L**,**P**) Magnified images showing CD-31 staining in the cells lining the perforations in the transition zone. (**A**–**D**) Valve in the early second trimester (111 days into gestation); (**E**–**H**) late second trimester (163 days into gestation); (**I**–**L**) third trimester (219 days into gestation); (**M**–**P**) full term (270 days). CD-31 positive cell staining is shown in brown. Cell nuclei are stained blue. Scale bar and magnification vary per image.

Positive staining for the endothelial cell marker CD-31 was observed, as expected, at the outer endothelial lining [41] of the anterior leaflet (Figure 7B,F,J,N) and chordae tendineae (Figure 7C,G,K,O). Interestingly, cells lining the transition zone perforations were also positive for CD-31 staining in every age group examined (Figure 7D,H,L,P), suggesting these cells possess an endothelial cell identity.

### 3.4. MMP-1 and MMP-2 Are Present Around Endothelial Cells Lining the Transition Zone Perforations

**Figure 8:**
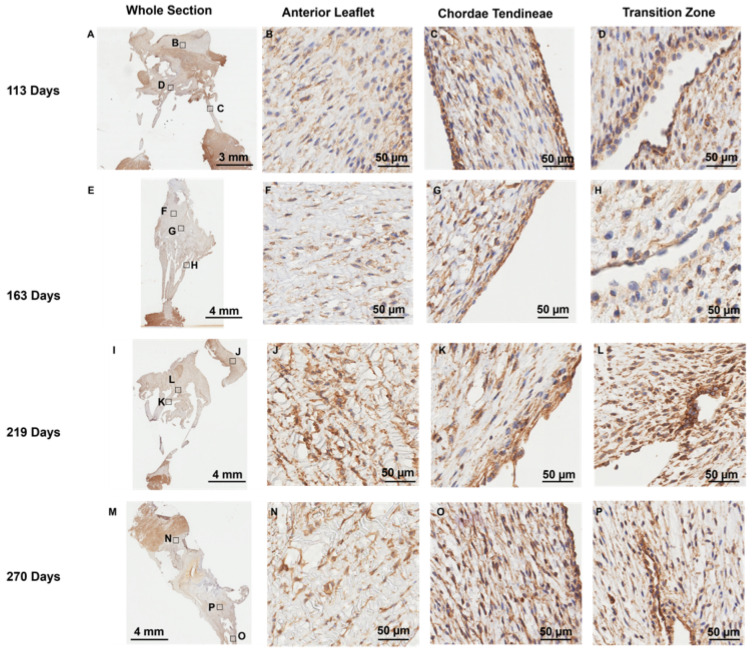
Representative images of MMP-1 expression throughout the mitral valve anterior apparatus. (**A**,**E**,**I**,**M**) Overview of the MMP-1-stained sections. Boxes show where subsequent magnified panel images were taken from. (**B**,**F**,**J**,**N**) Magnified images of the body of the anterior leaflet showing diffuse MMP-1 staining throughout. (**C**,**G**,**K**,**O**) Magnified images of the chordae tendineae showing diffuse MMP-1 staining within the chordae and dense expression in the outer chordal edges. (**D**,**H**,**L**,**P**) Magnified images showing MMP-1 staining in the cells lining the perforations in the transition zone as well as the tissue around them. (**A**–**D**) Valve in the early second trimester (111 days into gestation); (**E**–**H**) late second trimester (163 days into gestation); (**I**–**L**) third trimester (219 days into gestation); (**M**–**P**) full term (270 days). MMP-1 positive staining is shown in brown. Cell nuclei are stained blue. Scale bar and magnification vary per image.

**Figure 9:**
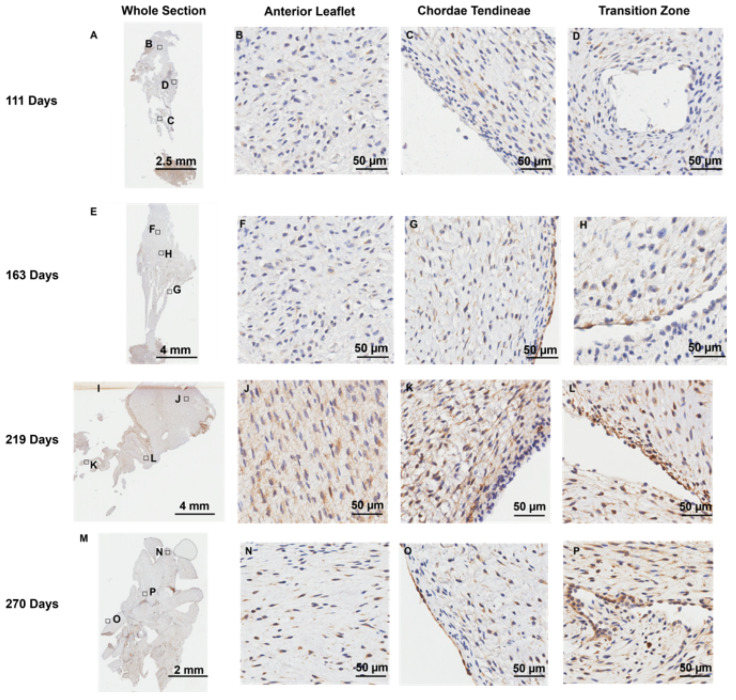
Representative images of MMP-2 expression throughout the mitral valve anterior apparatus. (**A**,**E**,**I**,**M**) Overview of the MMP-2-stained sections. Boxes show where subsequent magnified panel images were taken from. (**B**,**F**,**J**,**N**) Magnified images of the body of the anterior leaflet showing diffuse MMP-2 staining throughout. (**C**,**G**,**K**,**O**) Magnified images of the chordae tendineae showing diffuse MMP-2 staining within the chordae and dense expression in the outer chordal edges. (**D**,**H**,**L**,**P**) Magnified images showing MMP-2 staining in the cells lining the perforations in the transition zone as well as the tissue around them. (**A**–**D**) Valve in the early second trimester (111 days into gestation); (**E**–**H**) late second trimester (163 days into gestation); (**I**–**L**) third trimester (219 days into gestation); (**M**–**P**) full term (270 days). MMP-2 positive staining is shown in brown. Cell nuclei are stained blue. Scale bar and magnification vary per image. Note that to preserve tissue integrity, the epitope retrieval time was reduced for MMP-2 staining; therefore, differences in intensity cannot be interpreted as differences in the levels of MMP expression between MMP-1 and MMP-2 sections.

Positive staining for MMP-1 (Figure 8) and MMP-2 (Figure 9) was diffused throughout the whole anterior mitral apparatus over gestation. However, the most intense MMP staining was in the vicinity of the cells that expressed CD-31 at the inner lining of the transition zone perforations (MMP-1: Figure 8D,H,L,P, MMP-2: Figure 9D,H,L,P) and at the outer edge of the chordae (MMP-1: Figure 8C,G,K,O, MMP-2: Figure 9C,G,K,L).

### 3.5. Cells Lining the Transition Zone Perforations Are Also Undergoing Proliferation

**Figure 10:**
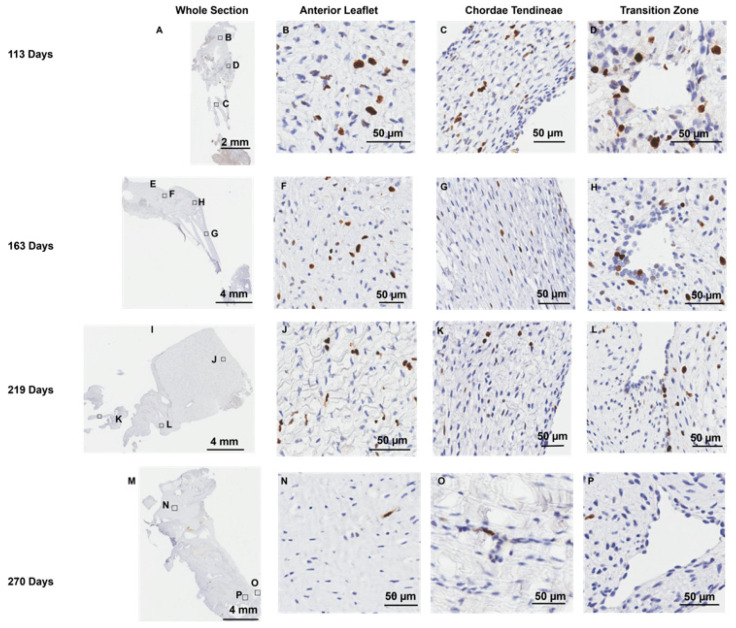
Representative images of Ki-67 expression throughout the mitral valve anterior apparatus. (**A**,**E**,**I**,**M**) Overview of the Ki-67-stained sections. Boxes show where subsequent magnified panel images were taken from. (**B**,**F**,**J**,**N**) Magnified images of the body of the anterior leaflet showing Ki-67 positive cells. (**C**,**G**,**K**,**O**) Magnified images of the chordae tendineae showing Ki-67 positive cell staining. (**D**,**H**,**L**,**P**) Magnified images showing Ki-67 staining in the cells lining the perforations in the transition zone as well as in the tissue around them. (**A**–**D**) Valve in the early second trimester (113 days into gestation); (**E**–**H**) late second trimester (163 days into gestation); (**I**–**L**) third trimester (219 days into gestation); (**M**–**P**) full term (270 days). Ki-67 positive cells are shown in brown. Ki-67 negative cell nuclei are stained blue. Scale bar and magnification vary per image.

**Figure 11:**
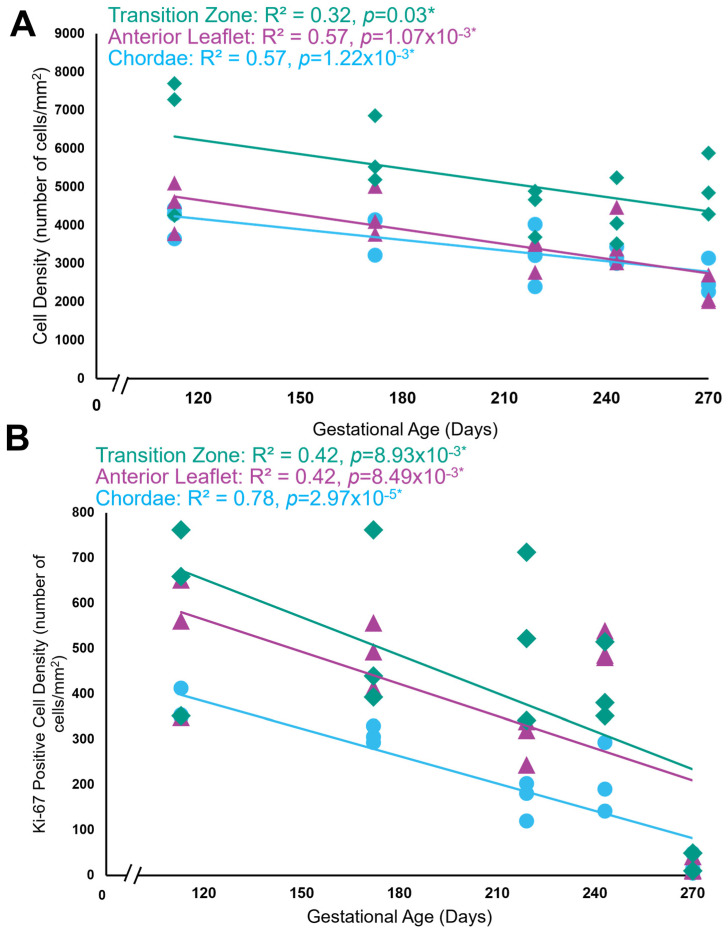
Both total cell density and total Ki-67 positive cell density decreased over time but were highest within the transition zone. (**A**) Total cell density (number of cells per mm^2^) in the anterior leaflet (purple triangles), the chordae tendineae (blue circles), and the transition zone (green diamonds) plotted as a function of gestational age, showing a significant correlation between these variables for each area. (**B**) Total Ki-67 positive cell density (number of cells per mm^2^) in the anterior leaflet (purple triangles), the chordae tendineae (blue circles), and the transition zone (green diamonds) plotted as a function of gestational age, showing a significant correlation between these variables for each area. Data are shown from five animals of five gestational ages. For each animal, data are shown for the anterior leaflet, the transition zone, and chordae tendineae, where three grids were analyzed in each region. Note the *x*-axis break on both graphs. * Denotes a slope significantly greater than zero.

Positive staining for the cell proliferation marker Ki-67 was evident throughout gestation in the anterior leaflet (Figure 10B,F,J,N), the transition zone (Figure 10D,H,L,P), and the chordae tendineae (Figure 10C,G,K,O). The transition zone had the highest density of proliferating cells, most often localized to the larger rounder cells lining the transition zone perforations (Figure 10D,H,L,P). Both total cellular density (Figure 11A) and Ki-67 positive cell density (Figure 11B) decreased over gestation throughout the whole anterior mitral apparatus, reflecting a decrease in cellularity and proliferation during fetal development.

### 3.6. Clusters of Proliferating Cells Expressing MMPs Are Present Within the Transition Zone

**Figure 12:**
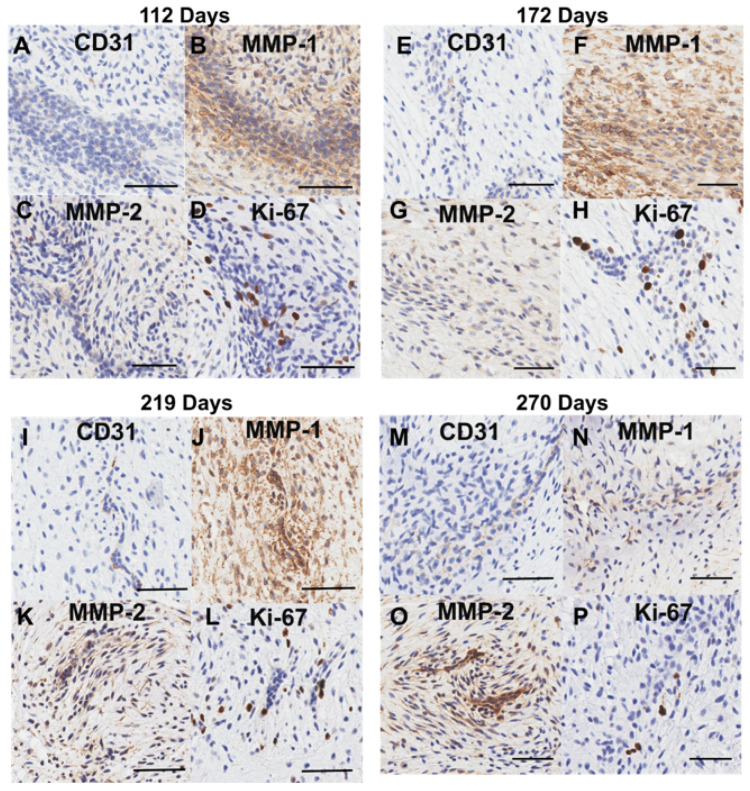
Representative images of the cell clusters present within the transition zone throughout gestation. (**A**,**E**,**I**,**M**) CD-31staining is present in cells within the clusters. (**B**,**F**,**J**,**N**) MMP-1 staining is found within and around the cell clusters. (**C**,**G**,**K**,**O**) MMP-2 staining is found within and around the cell clusters. (**D**,**H**,**L**,**P**) Some cells within the clusters are positive for Ki-67 staining. (**A**–**D**) Valve in the early second trimester (113 days into gestation); (**E**–**H**) late second trimester (163 days into gestation); (**I**–**L**) third trimester (219 days into gestation); (**M**–**P**) full term (270 days). Positive marker staining is shown in brown. Cell nuclei are stained blue. The scale bar varies per image.

Contributing to the high cell density in the transition zone were large clusters of cells (Figure 12). Cells within these clusters were positive for CD-31 (Figure 12A,E,I,M), MMP 1 (Figure 12B,F,J,N), MMP 2 (Figure 12C,G,K,O), and Ki-67 (Figure 12D,H,L,P), suggesting that these are clusters of proliferating cells (potentially endothelial in origin)—*within the bulk of the transition zone tissue*—that are remodeling via proliferation and ECM degradation.

### 3.7. Transition Zone Perforations Are Also Present in 3D Imaging

**Figure 13:**
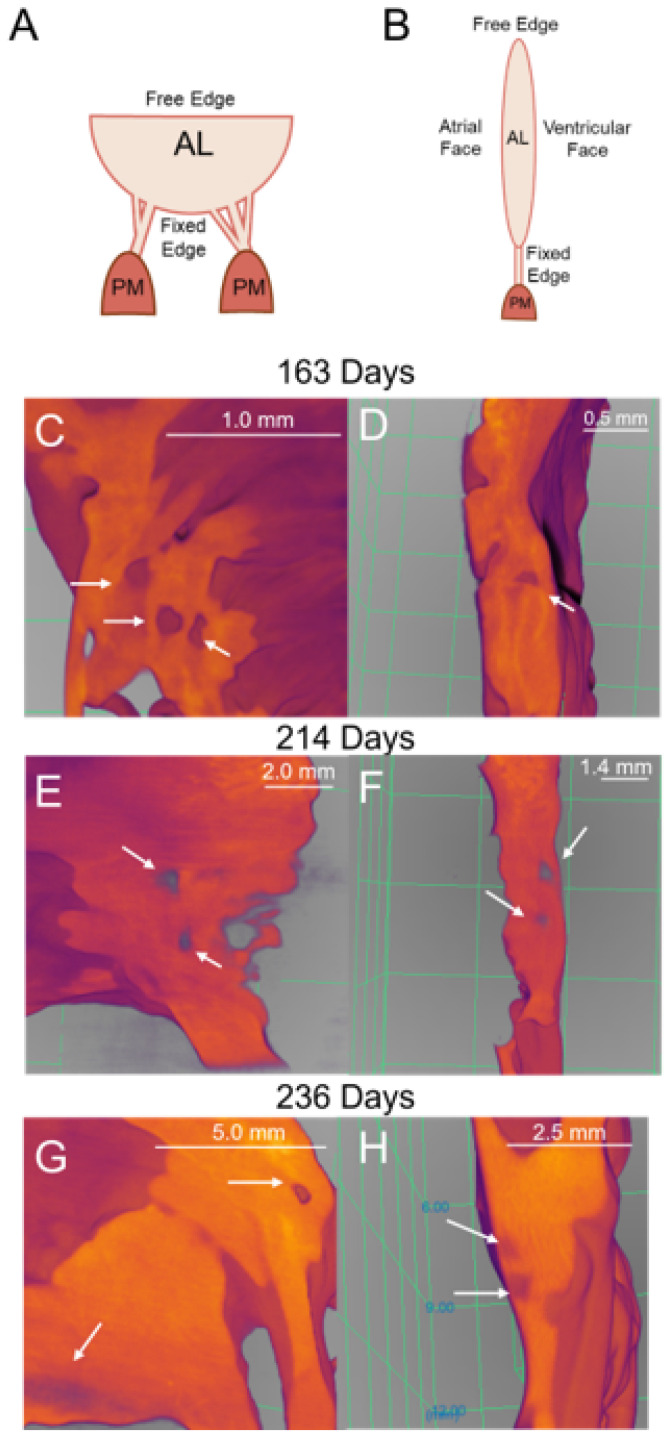
Similar perforations to those found in histology are present in micro-CT 3D renderings of the fetal mitral valve. (**A**) Diagram representing the orientation of the mitral valve in (**C**,**E**,**G**) where the view is of the ventricular face. (**B**) Diagram representing the orientation of the mitral valve in (**D**,**F**,**H**) where it is a side view of the mitral valve. (**C**,**D**) Late second trimester (163 days into gestation) ventricular view (**C**) and side view (**D**). (**E**,**F**) Third trimester (214 days into gestation) ventricular view (**E**) and side view (**F**). (**G**,**H**) Later third trimester (236 days into gestation), ventricular view (**G**) and side view (**H**). White arrows denote perforations, seen as distinct circular disruptions in the structure. Image color is related to optical density; bright orange is denser tissue. The scale bar varies per image.

Perforations observed in micro-CT imaging and histology were the same order of magnitude in dimensions, suggesting these were the same structural features in both modalities. Different ECM proteins cannot be differentiated in the 3D renderings, but perforations could be visualized in the transition zone tissue (Figure 13). As well, smaller areas where the tissue was thinner were also notable in the transition zone (Figure 13G).

**Figure 14:**
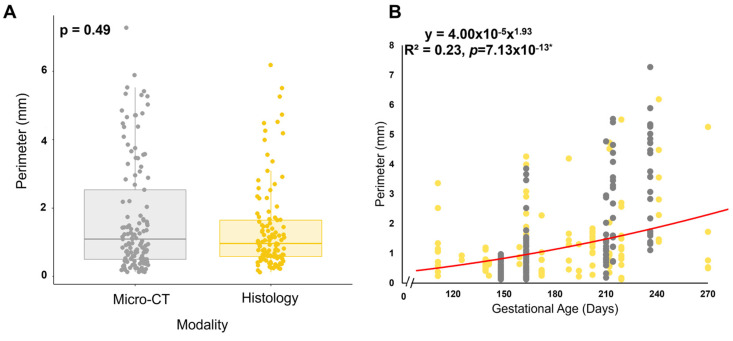
Observed perforations possess similar dimensions between both imaging modalities. (**A**) A Mann–Whitney U test determined there was no significant difference. (**B**) Perimeter (mm) versus gestational age (in days) demonstrating a strong relationship between these variables. As there was no significant difference, all the perimeter sizes were binned together for the power relationship analysis. * and solid red line denote a slope significantly greater than zero. Each point represents *n* = 1 perforation. The total sample size was *n* = 44 for micro-CT and *n* = 56 for histology.

To determine if the perforations observed in the micro-CT images corresponded to the cell-lined perforations observed in the histology sections, the perimeters of each perforation were measured in both modalities (Figure 2). A Mann–Whitney U test demonstrated that there was no significant difference in the dimensions of the perforations between both modalities (*p* = 0.49, Figure 14A). Interestingly, the size of the perforations increased with gestational age, demonstrating a strong relationship (Figure 14B).

### 3.8. A Continuous Population of Collagen Fibrils Run Between the Trunk and Branch Chordae

**Figure 15:**
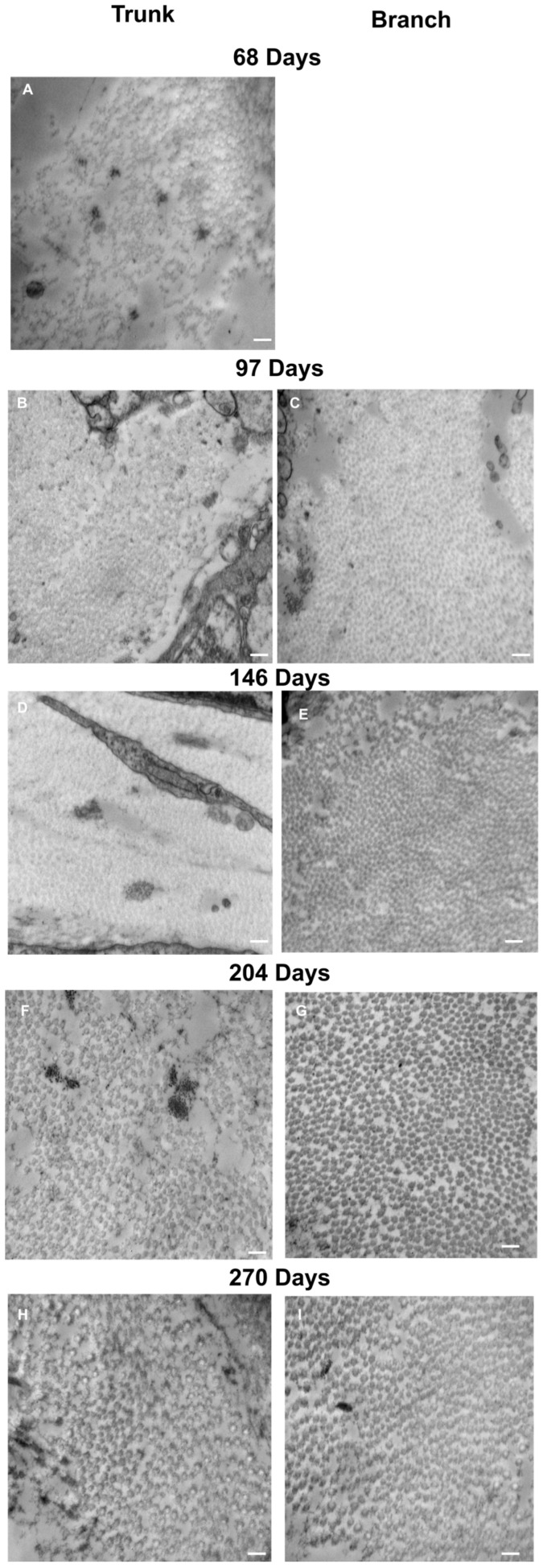
Representative transmission electron microscopy images of the trunk (**A**–**E**) and branch chordae (**F**–**I**) over gestation. (**A**) First trimester (68 days into gestation), no discernable branches were apparent. (**B**,**F**) Early second trimester (97 days into gestation). (**C**,**G**) Mid second trimester (146 days into gestation). (**D**,**H**) Early third trimester (204 days into gestation). (**E**,**I**) Full term (270 days into gestation). All images were taken at 50,000× magnification. The scale bar represents 200 nm.

Transmission electron microscopy was performed to image collagen fibrils in cross-section from chordae branches and their common trunk (Figure 3). An increase in collagen fibril diameter over gestation is visible in the chordae branch and trunk (Figure 15).

**Figure 16:**
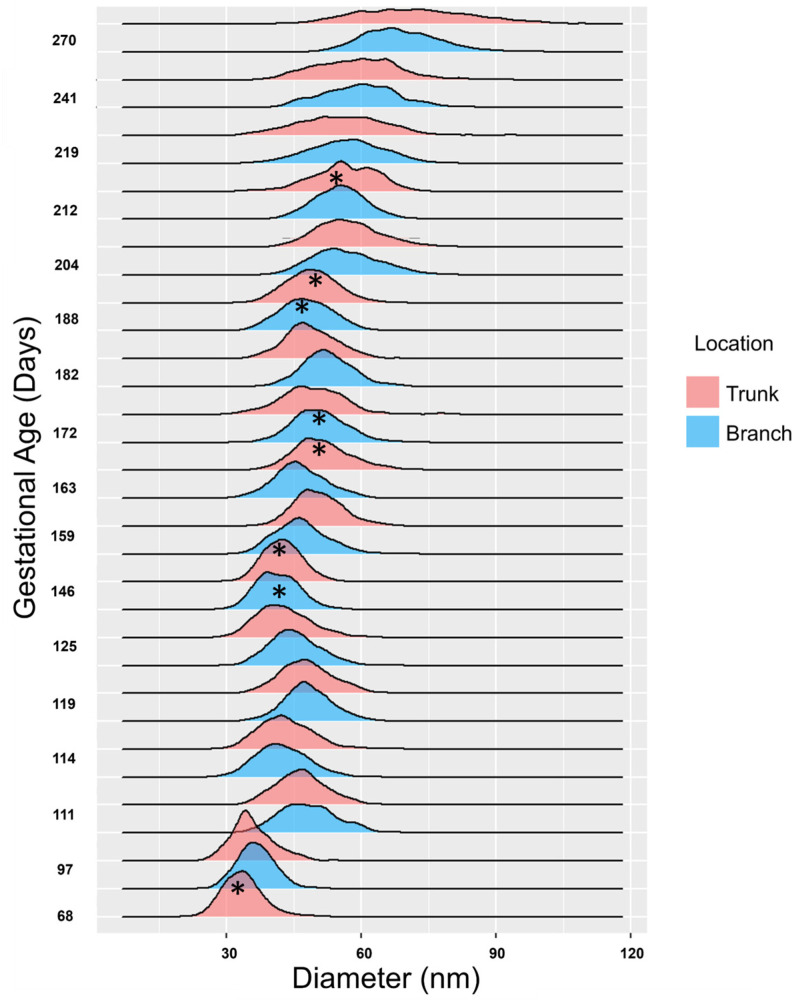
Collagen fibril diameters increase over gestation, with distributions alternating between unimodal and bimodal distributions in both the trunk and branch chordae. Collagen fibril diameters (in nm) are plotted as a histogram and binned by both gestational age in days as well as location (trunk in red, branches in blue). * denotes a bimodal distribution as measured via Hartigan’s diptest. Note that at 68 days, there were no discernable branch chordae, so only the trunk is shown.

Collagen fibril diameters were measured and plotted as separate distributions for each branch and trunk pair at each gestational age examined (Figure 16). For both branch and trunk chordae, fibril diameter distributions demonstrated a rightward shift to larger fibril diameters over gestation (Figure 16).

**Figure 17:**
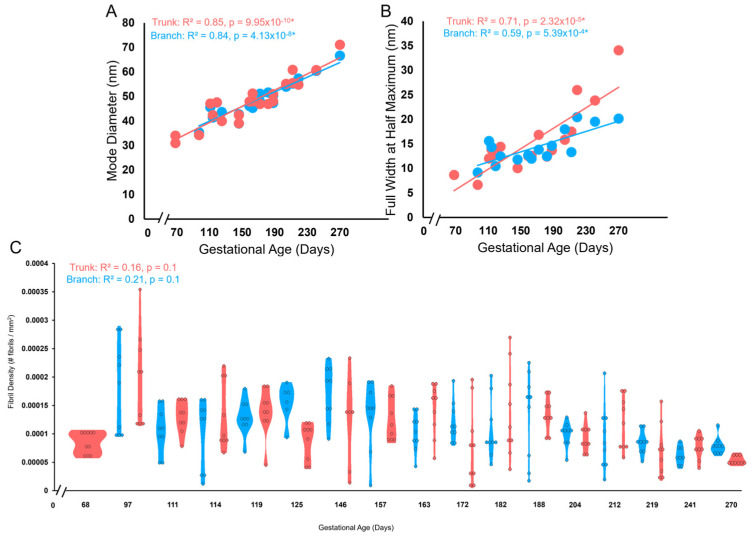
The trunk and branch chordae exhibit similar increases in the collagen fibril diameter and FWHM with a constant collagen fibril density over gestation. (**A**) Mode diameter of the trunk (red circles) and branches (blue circles) in nm plotted as a function of gestational age. (**B**) FWHM of the trunk (red circles) and branches (blue circles) in nm plotted as a function of gestational age. There was no difference in the rate of increase between branches and trunk as measured by ANCOVA. (**C**) Violin plot of fibril density (number of fibrils per mm^2^) versus gestational age in days for either the trunk (red) or branches (blue). * denotes a slope significantly greater than zero.

Bimodality appeared in the distributions in the first trimester, then shuffled between bimodal and unimodal thereafter in both branch and trunk chordae (Figure 16 and Figure 17A). Contrary to bimodality in collagenous adult structures (with one large mode and one smaller mode), the fetal chordae modes were typically close together (e.g., 30.9 nm and 34.0 nm at 68 days). Fibril diameter modes increased over gestation in branch and trunk chordae, with no difference in their rate of increase (Figure 17A). The full width at half maximum (FWHM) was used to evaluate the heterogeneity of diameters within the chordae (Figure 4). The FWHM increased over gestation in both branch and trunk chordae with no difference in their rate of increase (Figure 17B). In contrast to fibril diameters, fibril density remained constant over gestation in both the branch and trunk chordae (Figure 17C). Taken together, the TEM results reveal similar collagen fibril populations in the branch and trunk chordae, suggesting that a common population of fibrils runs from the branch into the trunk.

## 4. Discussion

**Figure 18:**
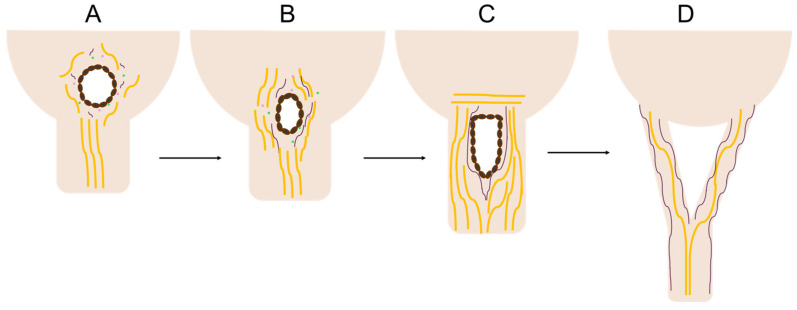
Schematic depicting the stages of perforation development that splits a single attachment into two. (**A**) Perforations created by endothelial cell clusters possess an internal lining of endothelial cells (brown ovals with blue nuclei) that express MMP-1 (pink circles) and MMP-2 (green circles). Shorter collagen fibers (yellow) and elastic fiber fragments (purple) are found surrounding the perforation. (**B**) Collagen and elastic fibers begin to align in parallel with the chordae axis. (**C**) The cellular activity splits the single chordal attachment site into two, with collagen and elastic fibers aligning in parallel with the chordal axis. (**D**) The split continues to propagate longitudinally, creating two new chordal branches, and the perforation is remodeled into the bifurcation region.

This study has revealed a unique region of cellular activity and ECM arrangement at the junction between the fetal mitral valve anterior leaflet and chordae tendineae. This “transition zone”, situated between collagen-dense areas of the leaflet and chordae, contains a region primarily composed of proteoglycans and features localized tissue thinning, endothelial cell-lined perforations, and clusters of proliferating cells. We hypothesize that this zone represents a region where chordae tendineae bifurcate during fetal development. In particular, perforations created by localized MMP activity from cell clusters (possibly endothelial in origin) serve as a site for the initiation of a “split” of a single chordae attachment into two (Figure 18A–C). The split propagates between collagen bundles into the preexisting chordae trunk, traveling towards the papillary muscle, creating two branches and the bifurcation region (Figure 18D).

A similar perforated region has been noted in the developing mitral valve structures across different species, including rats [15], chicks [27], and humans [21,22]—although much earlier in gestation—where the primordial anterior leaflet is connected directly to the papillary muscle. In rats (where chordae bifurcate postnatally), Masson’s trichrome staining demonstrated that this area was also comprised of predominantly proteoglycans with perforations in the tissue [15]. Scanning electron microscopy in developing chicks revealed depressions in this region, followed by perforations later in gestation [27]. The authors suggested these depressions and perforations were lined with endothelial cells based on morphology [27]. The present study confirms the endothelial identity of these transition zone cells. In early human mitral valve development, similar cell-lined perforations in the tissue were observed just above the papillary muscle [21]. In each study, the authors note that these depressions and perforations in the tissue precede the development of branched chordae tendineae [15,21,27]. Taken together the results from our study and the literature suggest that this perforated zone may be the area where new chordae tendineae branches are formed.

**Figure 19:**
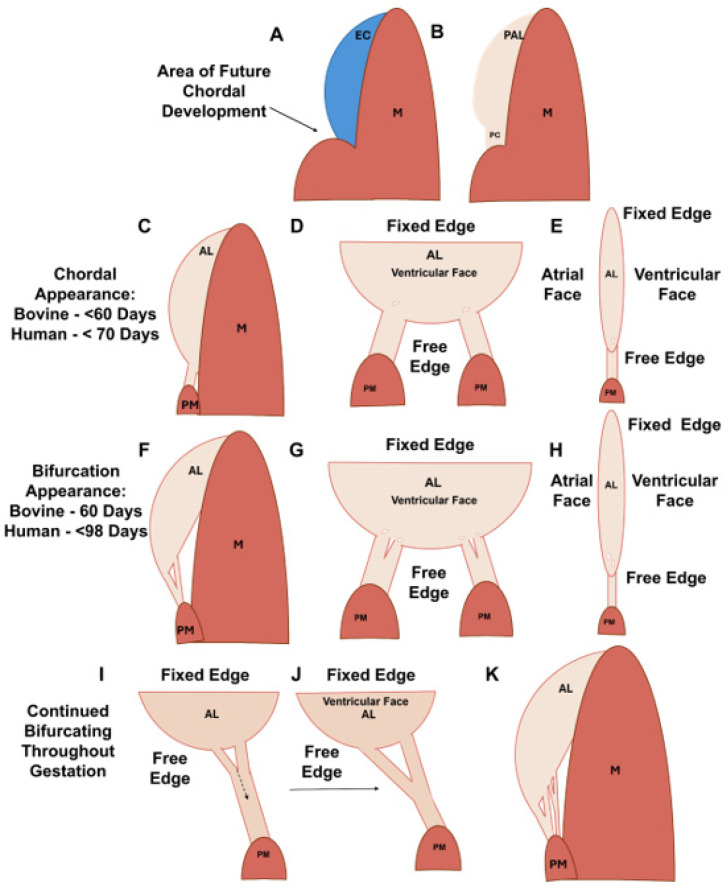
Schematic depicting the stages of chordae tendineae formation over gestation. (**A**) In the post-fusion endocardial cushion (EC), the area that will form the first chordal tendon is specified towards the papillary muscle connection. (**B**) After cushion remodeling, the primordial anterior leaflet (PAL) possesses a more semilunar shape, and the primordial chordae (PC) is a thin section of tissue connected to the PAL and the papillary muscle. (**C**) Sometime before 60 days in bovines (before ~70 days in humans), the PC has been remodeled into the first chordal tendons while the anterior leaflet (AL) remains connected to the underlying myocardium (M). (**D**) The ventricular face view at the same time point showing the first two chordae with perforations forming at their attachment points. (**E**) The side view at the same time point showing the perforation. (**F**) The first bifurcation appears at approximately 60 days in bovines (sometime before 98 days in humans) as the anterior leaflet has begun to delaminate from the underlying myocardium. (**G**) The ventricular face view at the same time point showing the first initial branches and subsequent perforation formation to further bifurcate the new branches. (**H**) The side view at the same time point showing subsequent perforation formation. (**I**,**J**) The initiated split from the perforations will continue to propagate longitudinally down the trunk toward the papillary muscle (PM). (**K**) The end result of fetal development is a fan-like network of attachments to the leaflet.

Using our observations and those from the literature, we can put together a more complete picture of chordae formation and bifurcation in a large animal model. Chordae formation begins in the post-fusion endocardial cushion with the specification of cells that will contribute to either leaflet or chordal development (Figure 19A). These two populations display distinct cellular phenotypes and protein expression profiles [19,42]. In mice and chick models, leaflet forming cells, responsive to bone morphogenetic protein 2 (BMP2), induce the expression of the cartilage markers sox9 and aggrecan, whereas chordae forming cells, responsive to fibroblast growth factor 4 (FGF4), induce the expression of tendon markers scleraxis and tenascin [42].

The first chordal tendon forms at the interface between the primordial leaflet and the papillary muscle in the first trimester [21] (Figure 19B,C). In chicks [27] and humans [21,22], the endocardial cells forming the chordae transform the cushion papillary interface into a thinner sheet that becomes the first chordal tendon (Figure 19B). This tendon remodels to attain its characteristic cylindrical shape (Figure 19C), likely via MMP-1 (Figure 8C,G,K,O) and MMP-2 (Figure 9C,G,K,O) expression at the outer chordae surface that persists over gestation.

In the mid to late first trimester [21,22,23], branching starts with the first chordal tendon (Figure 19D,E). Localized clusters of cells in the transition zone express MMP-1 (Figure 12C,G,K,O) and MMP-2 (Figure 12D,H,L,P), degrading the collagen and proteoglycan substrate [43], thinning the tissue and creating perforations. Double immunostaining for both CD31 and MMPs are needed to confirm if the cells secreting MMPs are endothelial in origin. As well, RNA detection for MMPs would be beneficial for confirming their localization throughout development. As gestation progresses, further bifurcations are created with the same process (Figure 19F–H).

Transition zone collagen and elastic fibers, initially present as sparse, short fragments, begin to condense around the perforations (Figure 6A–C and Figure 18A–C) beginning around the second trimester. Fibers then rotate into the radial direction of the valve towards the papillary muscle (Figure 6A–C and Figure 18B), where they will become incorporated into the newly formed chordal branches (Figure 18C,D). Collagen fibers become localized in the bulk of the new branches, with dense, nearly continuous elastic fiber layers at their periphery (Figure 18D). This is consistent with our earlier observation of the elastin “jacket” in fetal chordae becoming fully developed in late gestation [23]. Endothelial cells within and around the perforations proliferate to provide new cells to line the outer edges of the newly formed chordae branches (Figure 10D,H,L).

The synthesis of elastic fibers in the transition zone may play important signaling roles in chordal branching. Elastin peptides have been shown to decrease endothelial cell proliferation [44]. Therefore, as elastogenesis increases in the transition zone in late gestation, it may trigger a decrease in endothelial cell proliferation activity and an end to chordal branching. Indeed, by full term, cell proliferation has decreased with fewer Ki-67 expressing cells lining the perforations (Figure 10P) and in the bulk of the transition zone tissue (Figure 11B and Figure 12P), suggesting that chordae bifurcation is slowing. This is in line with our previous work that suggests that the number of chordae attachments to the leaflet is set at birth [23]. Double immunostaining for both CD31 and Ki-67 is needed to confirm if the proliferating cells are indeed endothelial in origin.

Mechanical loading plays several key roles in valvulogenesis [45] and undoubtedly in chordae tendineae bifurcation. For example, when filamin-A—a mechanosensory component of valvular endothelial cells—is knocked out in mice, chordae tendineae branching does not occur [24]. The loss of mechanosensing abilities may disrupt the ability of the cells to create perforations and ultimately inhibit chordal branching. There may also be an interplay between mechanical loading and the transition zone perforations during chordae bifurcation. A hole in a tissue under tension concentrates stresses in the adjacent tissue, causing the hole to stretch more than tissue far from the defect [46]. Elevated stress around the perforations may trigger the accumulation of collagen and elastic fibers in those regions (Figure 6). Indeed, a similar process occurs during arterial growth, where holes or “fenestrations” in the elastic lamellae are created via the expression of MMP-2 [47,48]. Stress concentrations rapidly enlarge the fenestrae—contributing to the growth of the tissue—but also trigger the deposition of new elastic fibers around the hole circumference [47]. We see a similar parallel increase in perforation size and leaflet area, where both parameters exhibited a power relationship with gestational age. Perforations in the developing mitral leaflet may also serve to concentrate stress, reorient the ECM, and initiate the “split” of a single attachment into two branches.

We propose that the split propagates down a single chordal attachment via a cleavage plane—likely between major collagen bundles (Figure 19I,J). This is supported by the similar populations of collagen fibrils in the trunk and the branch, suggesting that they were once one population of collagen fibrils that had been separated by a cleavage plane. Studies pulling apart branched and unbranched chordal tissues to mechanically induce splitting should be conducted to test this longitudinal splitting hypothesis.

While this work was able to capture a large range of gestation in a large mammal, there are limitations to these kinds of studies. We did not have the ability to measure changes in physiological measurements (blood pressure, flow profiles, heart rate, etc.) over gestation. Therefore, we cannot correlate physiological changes with the tissue level changes observed in our study. As well, we described a process of organogenesis in the mitral valve but did not have the ability to watch this happen in live time. The development of tissue-engineered or organoid valve tissues would be beneficial for studying chordae formation in real time.

## 5. Conclusions

This study provides the most robust hypothesis to date on the formation and bifurcation of chordae tendineae in a large mammalian model. As adult valve pathologies are increasingly linked to errors in fetal development, studies such as these can aid our understanding of the underlying mechanisms in these processes. The proposed longitudinal splitting mechanism effectively explains the malformation of chordae tendineae in disease states. Clinicians have observed the presence of large, thick, unbranched chordal trunks in congenital mitral valve diseases, such as mitral hemi-arcade or undifferentiated chordae. In these cases, the affected valves exhibit thick, unbranched chordae [49]. Building upon the longitudinal splitting hypothesis, it can be inferred that these thick, unbranched chordae result from errors in the normal branching process, preventing the formation of the intricate fan-like patterns seen in healthy valves. Given that the only current treatment for valve disease is surgical replacement, understanding chordae tendineae formation is essential for innovating new treatments that could address the underlying causes of these conditions. As tissue engineering strategies increasingly adopt a more developmentally focused approach, understanding mitral valve formation, including chordae tendineae development, becomes critical. Similarly, the ability to recapitulate the chordae formation processes is essential for the development of valve replacements that can grow in pediatric patients.

## Figures and Tables

**Table 1:** Antibody specific differences in immunohistochemistry protocol.

Step	CD31 Staining	MMP-1 Staining	MMP-2 Staining	Ki-67 Staining
Heat-Induced Epitope Retrieval Time (Minutes)	40	40	30	30
Primary Antibody and Dilution	CD31 rabbit polyclonal (Abcam, Cambridge, UK, ab28364, 1:50)	MMP-1 rabbit monoclonal (Abcam ab52631, 1:40)	MMP-2 rabbit polyclonal (Abcam ab97779, 1:200)	Ki-67 rabbit monoclonal (Abcam ab16667, 1:200)
Primary Antibody Incubation Time (Minutes)	60	60	30	60
Polymer Solution Incubation Time (Minutes)	12	12	8	12
DAB Incubation Time (Minutes)	20	20	10	20

## Data Availability

All datasets will be made publicly available at the time of publication and will be accessible at the request of the corresponding author.
